# Musician presence and its effects on physiological and psychological well-being in live versus livestreamed concerts

**DOI:** 10.1038/s41598-026-38194-3

**Published:** 2026-02-09

**Authors:** Antonia S. Becker, Julia Peters, Mathijs S. van Schie, Julian Schaap, Koen van Eijck, Michael Berghman, Femke Vandenberg, Norman van Dartel, Hans Jeekel, Markus Klimek

**Affiliations:** 1 Department of Neuroscience, Erasmus Medical Center, Dr. Molewaterplein 40, 3015 GD Rotterdam, The Netherlands; 2 Department of Public Health, Erasmus Medical Center, Rotterdam, The Netherlands; 3 Department of Cardiology, Erasmus Medical Center, Rotterdam, The Netherlands; 4 Department of Arts and Culture Studies, Erasmus University, Rotterdam, The Netherlands; 5 Faculty of Arts, University of Groningen, Groningen, The Netherlands; 6 Rotterdam Philharmonic Orchestra, Rotterdam, The Netherlands; 7 Department of Anesthesiology, Erasmus Medical Center, Rotterdam, The Netherlands

**Keywords:** Music, Concerts, Well-being, Live music, Heart rate variability, Heart rate, Psychology and behaviour, Psychology, Cardiology, Medical research

## Abstract

Listening to music, whether live or prerecorded, can benefit health and well-being. Studies have shown that music can alleviate pain and anxiety, improve emotional well-being, and strengthen social connectedness. While research on live music is promising, direct comparisons with recorded settings—specifically in relation to the physical co-presence of performing musicians—are limited. In this randomized controlled pilot study (n = 130), we examined the effects of live music concerts (classical and pop) compared to a simultaneous cinematic livestream, in a parallel design. Psychological and physiological well-being were assessed through self-reported questionnaires and physiological measures, including heart rate (HR) and heart rate variability (HRV). In the live condition, participants reported greater appreciation for the music, felt more moved by the music as indicated by higher kama muta scores, and reported higher arousal and valence compared to the livestream condition. Using linear mixed models, we found that only the mean HR was significantly higher in the live group, whereas other HRV parameters did not show statistically significant differences. In conclusion, the “liveness”—in terms of the musicians being physical present—emerges as an important aspect shaping audience’s subjective experiences and physiological responses, particularly HR.

**Supplementary Information:**

The online version contains supplementary material available at 10.1038/s41598-026-38194-3.

## Introduction

Arts and music have been shown to promote health and well-being^1–4^. Listening to music, whether live or recorded, can benefit both physical and psychological health^5,6^. For example, music can help regulate emotions and alleviate symptoms of anxiety and stress^7,8^. Additionally, studies have demonstrated that music can reduce acute and chronic pain in patient populations^9,10^. The types of music studied in research varies in terms of its characteristics (e.g., harmony, tempo and pitch), source (e.g., recorded or live music performance) and setting (e.g., listening alone or in an audience), which arguably align with different effects on the listener^11,12^. Some studies focus exclusively on the effects of (recorded) music, while others examine live music performances, which involve not only the music itself, but also the interaction between the audience and performers, as well as among audience members^5,13,14^. However, direct comparisons between live and non-live listening contexts remain scarce, leaving it unclear to what extent the “liveness” impacts audience well-being^15,16^.

In this study we define “liveness” as the physical co-presence and real-time interaction between audiences and performing musicians. Liveness is then measured on a continuum, where a higher degree of liveness entails both temporal simultaneity and close physical proximity between audience and performer^17^. Conversely, the lower-liveness condition in our study preserves temporal simultaneity and a shared physical space among audience members but lacks the physical presence of the musicians.

This randomized controlled pilot study investigates the impact of live music concerts compared to a simultaneous cinematic livestream on physiological and psychological well-being. Because group music listening is known to enhance social well-being^18^, we assessed participants’ subjective experiences using a survey that included kama muta—a construct capturing the feeling of being “moved” while experiencing social or emotional connectedness^19–21^. To complement these subjective reports, we assessed heart rate (HR) and particularly heart rate variability (HRV), which calculates changes in beat-to-beat time intervals, and is used as an objective indicator of autonomic nervous system (ANS) functioning and general health^22,23^. Decreased HRV has been linked to poorer long-term health outcomes, whereas increased HRV is associated with improved self-regulatory capacity^24,25^. In the context of music listening, evidence suggests that music can modulate ANS activity and thereby consequently influence HRV, although the direction of this effect varies^26^.

The live and livestream groups each attended six performances featuring both popular and classical music characterized by low familiarity. Familiarity is known to significantly influence emotional responses to music^27^. However, given that familiarity varies across individuals, a team of musical experts selected various pieces assumed to have low familiarity in order to ensure a comparable level of (un)familiarity across participants. Within the chosen genres, the performances varied in musical accessibility ‒ that is, the degree to which the music could be easily processed both cognitively and affectively ‒ as prior research suggests that this influences both musical appreciation and emotional responses such as kama muta^16,28,29^.

The aims of this study are (1) to compare the psychological and physiological responses between the live and livestream group and (2) to investigate the relationships between the respective condition (live vs. livestream), psychological experience, and more objectively measured HR and HRV. We hypothesize that the live condition will positively influence the psychological experience and increase participants’ HRV compared to the livestream condition, due to increased “liveness”.

## Background

The definition of “liveness” varies but tends to be understood as emerging from *spatiotemporal presence*, where performers and audiences share the same space and time^30^. Important aspects of liveness are therefore *simultaneity*—the performance unfolding in the moment—and *co-presence*, where both audience and performer are physically present. Based on this definition, co-presence is a crucial factor in the notion of “liveness”, which can refer to the shared presence of performers and audience members, as well as the shared presence among audience members. In this study, we focus on the aspect of close physical proximity and real-time interaction between audiences and performing musicians. Within this context, it is important to recognize that the comparison made in this study ‒between live and lower-liveness condition ‒ lies on a continuum of the concept “liveness”^31,32^. From this perspective, “liveness” is not a fixed property but a concept shaped by technological infrastructures, cultural practices and audience expectations^33^.

In this study, co-presence is defined as comprising both *physical* and *social* components. Physical co-presence refers to the objective condition in which people occupy the same space and time, enabling unmediated sensory and embodied interactions that can produce measurable physiological effects^34^. Social co-presence, by contrast, refers to the psychological sense of “being with others,” including perceived agency, intentionality and mutual awareness^35,36^. While physical co-presence can enhance social presence through richer sensory cues and interaction opportunities, social presence can also arise in mediated contexts, such as livestreams. In our design, the live concert condition includes both physical and social co-presence, whereas the livestream preserves some social presence through temporal simultaneity but lacks performers’ physical presence, allowing us to examine how these components contribute to psychological and physiological responses.

The experience of music is not merely a passive process but actively engages sensory, motor and emotional systems. These systems interact bidirectionally with the music to create meaning, as emphasized by embodied music cognition theory^37^. This embodied perspective emphasizes that listening to music often involves action-perception couplings and physical engagement, which shape how musical experiences are felt and understood. Evidence on live music reception has shown that the collective experience of live music can synchronize physiological responses^38–40^, influence head movements and hormone levels^15,41^, and can lead to social-emotional experiences of “collective effervescence”^42^. Additionally, comparative research indicates that the “liveness” of music is an important factor, as it affects psychological and physical engagement differently compared to mediated or streamed music^5,15,16,21^. In this context, it is important to precisely consider which factors of liveness are responsible for the differences observed.

Ultimately, (live) music has the potential to enhance well-being, a broad concept encompassing physical, emotional and social dimensions, and applied across disciplines such as social science, philosophy and medical research, with definitions varying by field^43^. Psychological well-being includes factors such as positive affect, satisfaction and purpose, whereas physical well-being is linked to illness and mortality^44^. These types of well-being are rarely independent of each other; studies show that psychological and social experiences are closely linked to physical outcomes and vice versa^45,46^. For example, music can influence emotional valence, which can in turn reduce (physical) pain^47^. Emotional responses can also be reflected in HR and HRV, which serve as objective physiological markers and can capture aspects of embodied experience^40,48^. HRV patterns may vary depending on factors such as music genre, tempo, and whether the performance is live or recorded^13,49^. Examining the interaction between physiological and psychological factors, negative emotional states—such as stress and aversive moods—are linked to reduced HRV, reflecting an objectively “stressed” state^50,51^. Conversely, higher HRV has been associated with greater subjective well-being, a relationship mediated by emotion regulation^52^. Nonetheless, the complex interplay between HRV and more subjective psychological and emotional experiences remains insufficiently understood—particularly in the context of music reception.

## Methods

### Participants and study setting

For this randomized controlled pilot study, first-year students from the International Bachelor Arts and Culture Studies at Erasmus University Rotterdam were invited to participate (n = 130). No sample size calculation was conducted, as this study was designed as a pilot. The study was conducted on January 17th, 2024, at ‘De Doelen’, a well-known concert venue in Rotterdam (see Supplementary Fig. 1 for the ground plan). All participants read and signed an informed consent form before participation. The study received prior approval from the ESHCC Research Ethics Review Committee of Erasmus University Rotterdam (ETH2324-0080). The study was conducted in accordance with the Universities of the Netherlands’ code of conduct and complied with the principles of the Declaration of Helsinki^53^. The study design and reporting followed the CONSORT 2015 guidelines for randomized trials (Supplementary Table 1). Adults aged 18 years and older were eligible to participate. There were no specific exclusion criteria for participation. Surveys were administered in English, as the study program is taught in English. Additionally, given the low prevalence of heart arrhythmia in this young population, participants’ medical history regarding this condition was not assessed.

### Study design and randomization

**Fig. 1:**
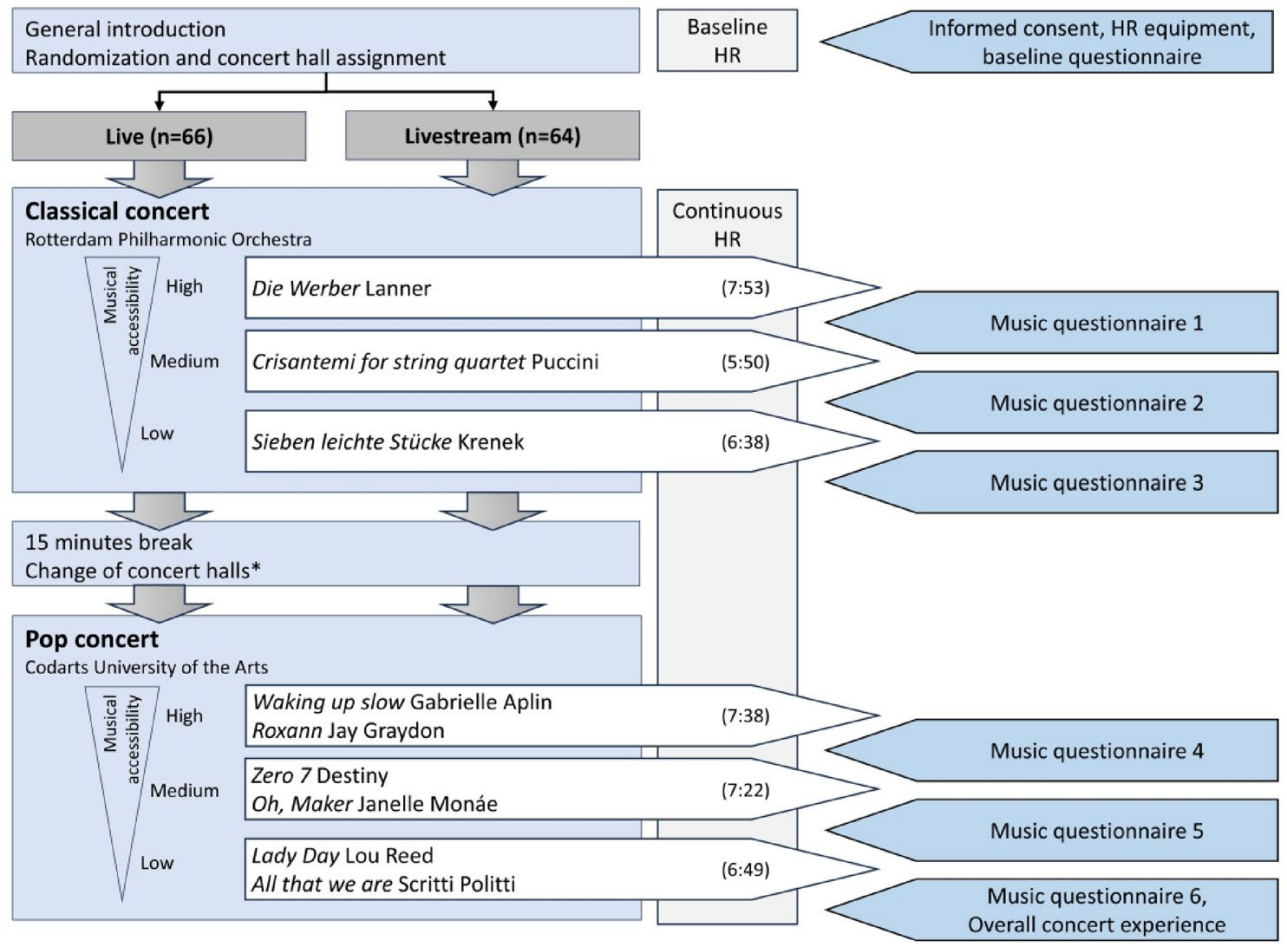
Study design. The figure illustrates the steps that both study arms simultaneously underwent at the concert venue ‘De Doelen’ in Rotterdam. The baseline HRV measurement lasted 6 min. The music performances were selected based on different levels of musical accessibility (high, medium, low). The length in minutes of each performance is shown in brackets. *All participants were instructed to stand up and move during the 15-min sessions. The live group changed rooms, while the other group was seated again in the same room with the cinematic livestream. HR, heart rate.

In a parallel design, all participants attended two concerts (pop and classical music), which were not public for the general audience. Figure 1 shows a schematic overview of the study design. Participants were randomly assigned to one of two groups by the researchers using the RAND function in Microsoft Excel, in accordance with the Cochrane Handbook for Systematic Reviews of Interventions^54^. One group attended the live performance, while the other watched a simultaneous livestream in a separate room within the same concert venue. Randomization allocation was disclosed only after the baseline measurement. Both the live and livestream group were seated in professional concert rooms with comparable seating, lighting, and sound amplification. This setup ensured that the primary distinction between the venues was whether the music was performed live or broadcast via a cinematic screen. Blinding was not possible due to the nature of the intervention.

Upon arrival, all participants were equipped with a Polar H10 heart sensor and a paper-and-pencil survey. Before entering their respective concert halls, participants filled in a baseline questionnaire and completed a 6-min baseline HR measurement. Participants then attended the classical concert, followed by the pop concert. Between the two concerts, there was a 15-min break during which all participants were instructed to leave their seat for comparability reasons. The live group was relocated to a different room between the classical and pop performances due to the distinct technical requirements of the two music genres, which necessitated a different stage setup for the musicians. In contrast, the livestream group remained in the same room throughout (Supplementary Fig. 1). Both concerts consisted of three performances. The classical concert included three individual compositions. Due to the typically shorter length of pop songs the performance of the pop concert consisted of three times two similar (in terms of musical accessibility) songs, i.e., six in total. The performances ranged from 5:50 to 7:53 min each adding to a total concert duration of approximately 30 min (for both classical and pop). After each musical piece, participants completed a questionnaire assessing their evaluation and familiarity with the music, as well as the valence, arousal, and kama muta they experienced during the performance. Following the final concert, participants completed a questionnaire containing several questions evaluating their overall experience of the concerts and the experimental procedure. HR was measured continuously during the experiment.

### Music selection

The concerts featured performances by professional musicians from the Rotterdam Philharmonic Orchestra (classical) and Codarts University of the Arts (pop). Music pieces of lower familiarity among the general public were chosen to diminish the role of preexisting associations and ensure a comparable level of (un)familiarity across participants. Moreover, music pieces with varying musical accessibility (low, medium, high) were selected by professionals from the Rotterdam Philharmonic Orchestra and Codarts, as research indicates that level of musical accessibility influences appreciation and kama muta ratings^16,28,29^. According to Eisentraut^29^, musical accessibility has three levels: physical access (being physically able to hear/register the music), personal reception (whether the music is perceived as simple or challenging to understand and engage with), and participation (whether one can be a part of a musical performance). Here, we focus exclusively on personal reception. High musical accessibility means the music is perceived as being easily understood, tolerated, enjoyed and/or engaged with. Medium to low-level musical accessibility indicates that it is perceived as more challenging or “difficult” to understand, tolerate, enjoy and/or engage with.

### Questionnaires

Participants completed paper-and-pencil surveys throughout the experiment. Initially, they completed baseline questionnaires covering their social background, cultural capital, music preferences, music engagement, TRAIT anxiety, State-Trait Anxiety Inventory (STAI)-6 (Cronbach’s α 0.83), and self-assessment manikin (SAM) to measure valence (Cronbach’s α 0.73) and arousal (Cronbach’s α 0.98)^19,55,56^. After each performance, participants evaluated the music they heard (appreciation and familiarity) and reported their immediate feelings (SAM and kama muta). The decision to include only these items was made to avoid disrupting the natural flow of the music performances and to minimize any potential emotional burden associated with completing extensive questionnaires. The kama muta scale is a validated psychosociological tool that assesses the feeling of “being moved” in a manner that enhances experiences of social connectedness. This questionnaire has been widely employed in previous research on live music reception to capture audiences’ immediate socioemotional responses^16,20,21^. In this study, twelve items on a 5-point Likert scale (Cronbach’s α = 0.91), which is based on the validated KAMMUS-S and KAMMUS-2 measures, were used, as shown in Supplementary Table 2^20,21^. The two music pieces for each pop performance (e.g., pop 1a and 1b) were evaluated separately after each performance. The ratings were then averaged per participant for each of the three performances. Finally, upon completion of all music performances, participants reassessed their anxiety levels with the STAI-6 and provided feedback on their overall concert experience through additional questionnaires.

### Heart rate (HR) and heart rate variability (HRV)

Each participant was equipped with a Polar H10 heart sensor, and surface electrocardiogram measurements were recorded via a mobile application provided by BioCheck BV. Researchers ensured that participants’ phones were connected to the correct heart sensors and that the sensors were placed properly. Measurements were started individually before the baseline measurement and ran continuously. The clock time of each study procedure (e.g., start of baseline measurement, start of concerts) was noted to calculate the time intervals for each participant. Between concerts, participants’ phones were checked individually to ensure that heart rate recordings were still active, to minimize data loss.

From these surface electrocardiogram measurements, the RR-interval duration and clock time were extracted. Using these beat-to-beat RR-intervals, data were subsequently analyzed per time interval (baseline and six music performances) using Python (version 3.11). First, the data was preprocessed to exclude artifacts and identify non-sinus rhythm beats. Second, HR parameters and HRV, including time and frequency domain parameters, were calculated (Table 1). Welch’s method was used to compute an estimate of the power spectral density for the frequency domain parameters. Additionally, we corrected RMSSD and SDNN using the coefficient of variance (cvRMSSD and cvSDNN) since the mean RR-interval duration varied across music performances^57^. This was done by dividing each by the mean beat-to-beat RR-interval of the corresponding time period and then multiplying by 100.

**Table 1:** Overview of HR and HRV parameters.

Parameter	Description
HR (beats/min)	
Mean Minimal (Min) Maximal (Max)	Heart rate
Time domain	
RR (ms)	RR-interval duration
SDNN (ms)	Standard deviation of the interbeat intervals of normal sinus beats
RMSSD (ms)	Root mean square of successive differences between normal sinus rhythm beats
Frequency domain	
LF (ms2)	Power of the low frequency band (0.04–0.15 Hz)
HF (ms2)	Power of the high frequency band (0.15–0.40 Hz)
LF/HF-ratio	Ratio of LF to HF power

### Statistical analysis

Data analysis and validation were performed using R-Studio (version 2023.12.0) and R (version 4.3.2) with the following packages: dplyr, tidyr, ggplot2, nlme, lavaan and psych. The distribution of the data was assessed via data visualization techniques, including histograms, QQ-plots, and the Kolmogorov–Smirnov test. For continuous normally distributed data, the mean and standard deviation were calculated. For non-normally distributed data, the median and percentiles were utilized. Categorical variables were presented as percentages. Baseline characteristics were compared between the two groups (live vs. livestream) using Fisher’s exact test for categorical variables and Welch’s t-test for numeric values. The change in STAI-6, measured before and after the two concerts, and differences in baseline measurement in HRV and SAM were compared between the live and livestream groups using Welch’s t-test.

Linear mixed models (LMMs) were used to investigate the influence of the group (live vs. livestream) on the psychological (appreciation, arousal, valence, kama muta) and physiological (HR and HRV) outcomes while accounting for the sequence order of the consecutive measurements (baseline and six music performances). Compound symmetry correlation structure was used due to model fitting convergence issues encountered with more complex structures. Based on information criteria (Akaike’s information criterion (AIC) and Bayesian information criterion (BIC)) and the log-likelihood test, a random slope was included in the model, and the fixed effects structure was determined. Additionally, a mediation analysis was performed to examine the effect of the condition (live vs. livestream) on HR mean, with subjective experience (appreciation, valence, arousal) as a mediator. The ΔHR mean, adjusted for the baseline measurement, was used and a multilevel structure was applied to account for the nested data within each participant. Listwise deletion was used to handle missing data.

## Results

**Fig. 2:**
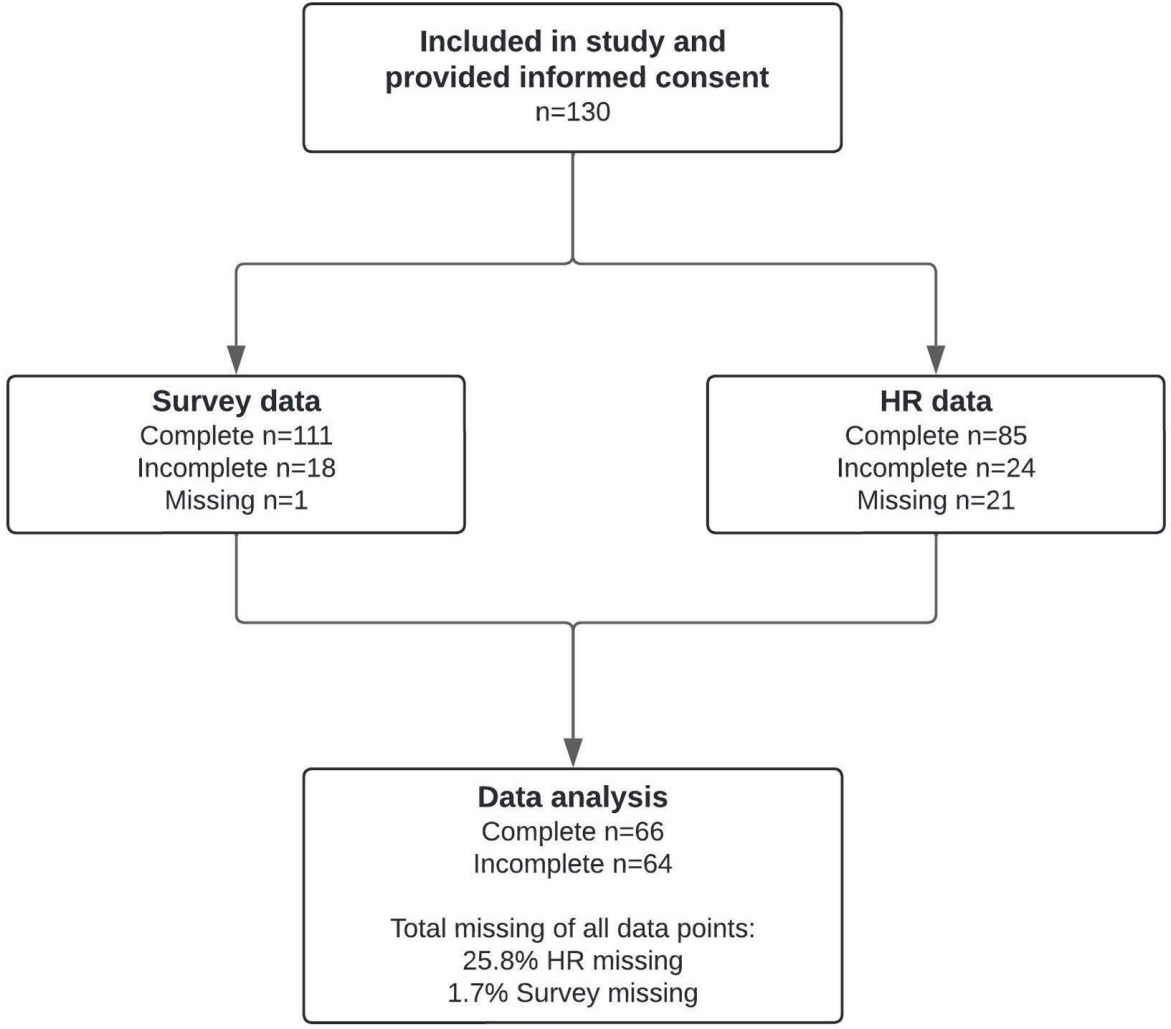
Overview of missing and included data in the analysis. Data were categorized as incomplete if at least one survey item or one HR data interval was missing. Missing and incomplete HR data resulted from either failed recordings or artifacts/non-sinus rhythms, which were consequently filtered out. In total, 25.8% of the HRV data and 1.7% of the survey data were missing from all the data points. HR, heart rate.

A total of 130 participants took part in the study, with 66 assigned to the live condition and 64 to the livestream condition. No participants were excluded in the enrollment process. However, there was a significant amount of missing HR data (25.8%), primarily due to technical problems (i.e. loss of connection between phone and chest strap). For one participant, the HR was recorded, but no survey data were available (Fig. 2). The study population consisted of first-year students from the Arts and Culture study program, who were relatively young, highly educated and had a strong interest in arts and culture. Table 2 presents the baseline characteristics for each randomized group. As we expected from this homogenous group, there were no significant differences between the groups after randomization. The live group tended to engage more regularly in exercise and sports, although this difference was not statistically significant. Additionally, participants’ familiarity with each music piece was assessed. This was relatively low, with an average of 13.4% of musical pieces being recognized by participants. This verifies our selection strategy aimed at minimizing prior familiarity with the musical pieces.

**Table 2:** Baseline characteristics of the study participants.

	Live (n = 66)		Livestream (n = 64)		*P*-value
	N	Value	N	Value	
Age (mean, SD)	65	22.45 (6.9)	64	21.72 (3.5)	0.451
Gender (n, %)	65		64		0.729
Female Male Other		54 (83.1) 8 (12.3) 3 (4.6)		49 (76.6) 13 (20.3) 2 (3.1)	
Dutch nationality (n, %)	64	25 (39.1)	60	17 (28.3)	0.256
TRAIT anxiety (mean, SD)	62	45.5 (10.2)	60	45.2 (10.6)	0.899
Appreciation of arts and culture* (mean, SD)	63	6.4 (0.7)	64	6.5 (0.6)	0.534
Genre rating (mean, SD)**					
Classical	65	3.7 (1.2)	63	3.7 (1.2)	0.927
Pop	65	3.9 (1.1)	63	3.9 (1.2)	0.959
Making music (n, %)	64	31 (48.4)	64	25 (39.1)	0.286
Visit a classical concert (n, %)	65		62		0.759
Never Once or twice a year Three to five times a year Once every month/two weeks Two to three times a month		20 (30.8) 32 (49.2) 6 (9.2) 6 (9.2) 1 (1.5)		21 (33.9) 25 (40.3) 10 (16.1) 5 (8.1) 1 (1.6)	
Visit a pop/rock concert (n, %)	65		62		0.998
Never Once or twice a year Three to five times a year Once every month/two weeks Two to three times a month		10 (15.4) 28 (43.1) 17 (26.2) 7 (10.8) 3 (4.6)		10 (16.1) 27 (43.6) 15 (24.2) 8 (12.9) 2 (3.2)	
Daily music listening (n, %)	65		64		0.940
Never Few minutes to half an hour Half an hour to an hour One to two hours Three to four hours Four hours and more		0 2 (3.1) 4 (6.2) 22 (33.9) 21 (32.3) 16 (24.6)		0 1 (1.6) 3 (4.7) 22 (34.4) 19 (29.7) 19 (29.7)	
Exercise or sports (n, %)	64		64		0.362
Never Once or twice a year Three to five times a year Once every month/two weeks Two to three times a month Once a week More than one a week		6 (9.4) 2 (3.1) 2 (3.1) 4 (6.3) 6 (9.4) 17 (26.6) 27 (42.2)		11 (17.2) 5 (7.8) 1 (1.6) 4 (6.3) 11 (17.2) 11 (17.2) 21 (32.8)	

Statistical significance was tested between the two groups (live vs. livestream) using Fisher’s exact test for categorical variables and Welch’s t-test for numeric values.

*7-point Likert scale.

**5-point Likert scale.

SD, standard deviation; TRAIT, Trait Anxiety Inventory.

### Psychological outcomes

**Fig. 3:**
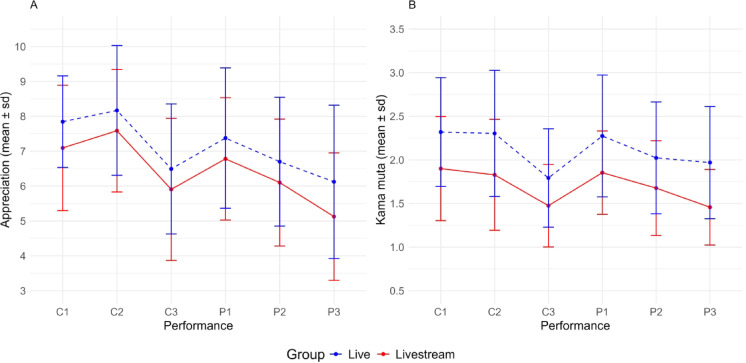
Appreciation and kama muta ratings per performance and randomization group. Appreciation (**A**) was assessed via a 10-point Likert scale. The mean score of the 12-item kama muta (**B**) scale (each 5-point Likert) was calculated. Mean and SD were calculated for each performance of the two concerts (C, classical, P, pop), which were performed in in the depicted order. SD, standard deviation.

**Fig. 4:**
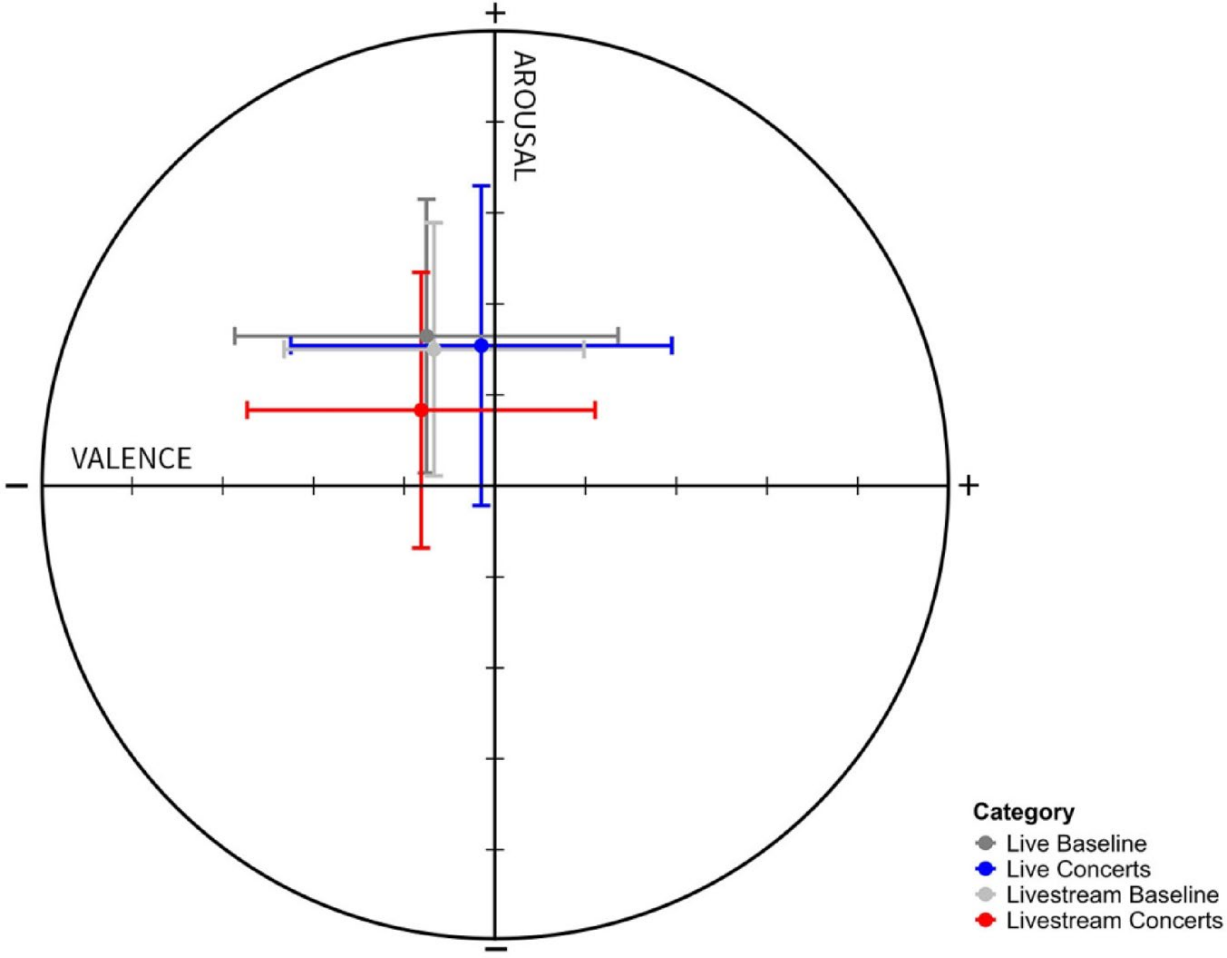
Arousal and valence ratings per group (live vs. livestream). Arousal and valence were measured on a 9-point Likert scale. The concert condition depicts the mean and standard deviation for all six performances.

Figure 3 presents an overview of the appreciation (10-point Likert scale) and kama muta rating (5-point Likert scale, 12 items averaged) of the six music performances. LMMs were conducted to compare the live versus livestream condition while considering the sequence order of the six performances. Classical 2 received the highest rating in terms of how much participants appreciated the music (livestream: mean 7.6 ± 1.8; live: mean 8.2 ± 1.9). In contrast, Pop 3 received the lowest rating in both groups (livestream: mean 5.1 ± 1.8; live: mean 6.1 ± 2.2). Notably, the live group consistently gave higher ratings for appreciation and kama muta (appreciation: β = 0.7; 95% CI 0.3 – 1.1, *p* = 0.001; kama muta: β = 0.2; 95% CI 0.3 – 0.6, *p* < 0.001; Supplementary Table 3). Moreover, an exploratory analysis of level of musical accessibility revealed a (statistically nonsignificant) trend, with greater appreciation observed at higher levels of musical accessibility. The live group rated their arousal and valence higher during all (classical and pop) music performances under consideration of the sequence order (arousal: β = 0.6; 95% CI 0.3 – 0.9, *p* < 0.001; valence: β = 0.6; 95% CI 0.1 – 1.0, *p* = 0.011; Supplementary Table 3; Fig. 4). The baseline measurements did not differ between both groups: arousal: livestream 6.5 ± 1.4, live 6.7 ± 1.5, *p* = 0.568; valence: livestream 4.3 ± 1.7, live 4.3 ± 2.1, *p* = 0.806. In other words, irrespective of music genre and level of musical accessibility, the live condition yielded higher scores on psychological parameters including appreciation, kama muta, valence and arousal compared to the livestream condition.

Before and after the concert, participants rated their state anxiety using the STAI-6. The scores decreased in both groups after the concert (livestream: before 37.1 ± 10.7, after 34.5 ± 8.8; live: before 38.4 ± 10.3, after 34.1 ± 8.5), with no significant differences between the live and livestream condition (livestream: Δmean − 3.1 ± 8.5; live: Δmean − 4.1 ± 11.6; *p* = 0.579).

### Physiological response

**Fig. 5:**
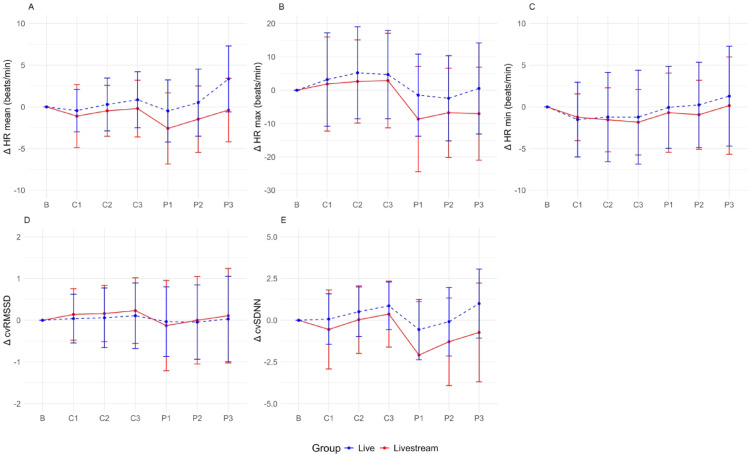
Centered time domain parameters per music performance. HR, heart rate; RMSSD, root mean square of successive differences; SDNN, standard deviation of NN intervals.

**Fig. 6:**
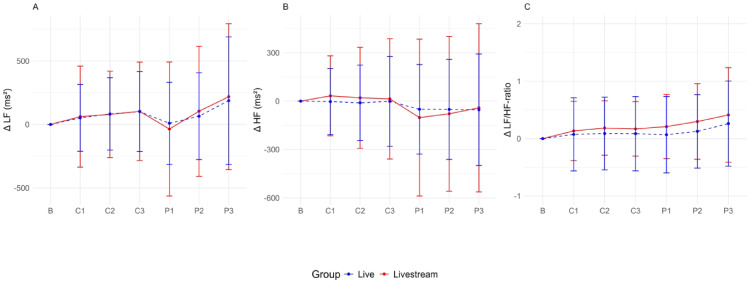
Centered frequency domain parameters per music performance. HF, high frequency; LF, low frequency.

Figures 5 and 6 present an overview of the centered HR and HRV parameters of the live and livestream groups. The baseline values were comparable between both conditions, showing no significant differences, with for example: HR mean (beats/min): livestream 78.96 ± 11.03, live 81.11 ± 10.19, *p* = 0.308; HR max (beats/min): livestream 110.80 ± 19.45, live 110.45 ± 14.06, *p* = 0.916; cvSDNN: livestream 13.37 ± 3.77, live 12.35 ± 3.14, *p* = 0.140; HF (ms^2^): livestream 913.32 ± 831.48, live 716.55 ± 554.40, *p* = 0.162. The impact of the live versus livestream condition on the HR and HRV parameters was tested, considering the sequence order of the baseline measurement and the six performances using LMMs (Table 3). Overall, the sequence order significantly affected LF (β = 22.55; 95% CI 5.10 – 39.99, *p* = 0.011), LF/HF ratio (β = 2.15; 95% CI 1.84 – 2.46, *p* < 0.001), HR min (β = 0.26; 95% CI: 0.06 – 0.45, *p* = 0.009) and HR max (β = -1.12; 95% CI -1.75 – -0.68, *p* < 0.001).

**Table 3:** Linear mixed model of HR and HRV parameters.

Parameter (Livestream n = 54, live = 52, with a total of 675 observations^a^)		Intercept	Sequence of music performances	Condition (0 = Livestream, 1 = Live)
HR mean (beats/min)	β (95% CI)	77.15 (71.31; 80.00)	0.14 (− 0.01; 0.30)	4.11 (0.09; 8.12)
	*p*-value	< 0.001***	0.071	0.045*
HR min (beats/min)	β (95% CI)	62.98 (60.33; 65.63)	0.27 (0.06; 0.45)	3.18 (− 0.54; 6.91)
	*p*-value	46.68***	0.009**	0.093
HR max (beats/min)	β (95% CI)	112.39 (107.50; 117.27)	− 1.21 (− 1.75, − 0.68)	3.69 (− 2.66; 10.04)
	*p*-value	< 0.001***	< 0.001***	0.251
cvSDNN	β (95% CI)	12.62 (11.76; 13.49)	− 0.08 (− 0.17; 0.02)	0.28 (− 0.81; 1.37)
	*p*-value	< 0.001***	0.108	0.612
cvRMSSD	β (95% CI)	5.37 (4.86; 5.88)	− 0.01 (− 0.04;0.03)	− 0.63 (− 1.34;0.08)
	*p*-value	< 0.001***	0.686	0.083
LF (ms2)	β (95% CI)	1481.40 (1216.11; 1746.68)	22.55 (5.10; 39.99)	− 350.37 (− 726.15; 25.42)
	*p*-value	< 0.001***	0.011*	0.067
HF (ms2)	β (95% CI)	941.25 (761.23; 1121.27)	− 12.94 (− 27.38; 1.51)	− 218.44 (− 460.67; 23.78)
	*p*-value	< 0.001***	0.079	0.077
LF/HF-ratio	β (95% CI)	2.15 (1.84; 2.46)	0.05 (0.02; 0.07)	− 0.21 (− 0.62; 0.21)
	*p*-value	< 0.001***	< 0.001***	0.328

Linear mixed models were conducted for all listed HRV parameters under consideration of the condition (0 = Livestream, 1 = Live) and the sequence order of the baseline measurement and six performances. The table depicts the fixed effects of the models. Based on information criteria (AIC and BIC) and log-likelihood test, a random slope for the sequence order was included in the model.

^a^Including the baseline measurement and the six consecutive music performances.

AIC, Akaike Information Criterion; BIC, Bayesian Information Criterion; cv, coefficient of variance; HR, heart rate; RMSSD, root mean square of successive differences; SDNN, standard; deviation of NN intervals; HF, high frequency; LF, low frequency; VLF, very low frequency.

*p < 0.05; **p < 0.01; ***p < 0.001.

Compared with the baseline measurement, the mean HR increased significantly more in the live condition during all music performances (β = 4.11; 95% CI 0.09 – 8.12, *p* = 0.045; Fig. 5a). Both groups showed a similar trajectory in terms of minimal HR, whereas the maximal HR showed a (statistically nonsignificant) trend toward higher values in the live group and during the classical performances (Fig. 5b and c). Because of the variability in mean HR during the music performances, the SDNN and RMSSD were corrected accordingly (cvSDNN, cvRMSSD). Both values showed different (statistically nonsignificant) trends, with the cvSDNN being higher in the live group and the cvRMSSD being marginally higher in the livestream group, with higher values indicating higher parasympathetic activity (Fig. 5d and e). The frequency domain parameters did not differ between the live and livestream groups (Fig. 6). Upon examination in an exploratory manner, some (statistically nonsignificant) trends were observed between the different music performances. The mean HR and cvSDNN values were higher for music pieces with lower musical accessibility (high: C1 and P1; medium: C2 and P2; high: C3 and P3), whereas the maximal HR showed higher values during the classical music performances. These trends were not further statistically tested because of the fixed sequence of musical accessibility level and music genre in the study design.

### Relationship between psychological and physiological outcomes

Next, we examined the interplay between participants’ psychological and physiological responses, assuming that the live versus livestream condition influences psychological reactions (appreciation, kama muta, arousal and valence), which, in turn, could affect physiological HRV measurements. Since only the mean HR significantly differed between the live and livestream groups when controlling for the sequence order of the baseline measurement and six performances, mediation analyses were conducted focusing on this parameter (Table 4; mediator model 1: appreciation; mediator model 2: kama muta, mediator model 3: arousal; mediator model 4: valence). The condition (live vs. livestream) significantly influenced all four psychological responses, with the live condition predicting higher values compared to the livestreamed condition. In all four models, the condition (live vs. livestream) also had a significant influence on the HR mean, with higher values observed in the live group compared to the livestream group. However, no effect of the mediators (appreciation, kama muta, valence, and arousal) on the HR mean was observed in all models.

**Table 4:** Mediation analysis of Δ HR mean and subjective experience.

Predictor	Outcome variable	Estimate (β)	SE	z-value	*p*-value
Model 1 (appreciation)					
Condition	Appreciation	0.67	0.24	2.84	0.005**
Appreciation	Δ HR mean (beats/min)	− 0.16	0.12	− 1.38	0.168
Condition	Δ HR mean (beats/min)	1.87	0.66	2.82	0.005**
Model 2 (kama muta)					
Condition	Kama muta	0.37	0.10	3.88	< 0.001***
Kama muta	Δ HR mean (beats/min)	− 0.29	0.37	− 0.77	0.439
Condition	Δ HR mean (beats/min)	1.86	0.66	2.81	0.005**
Model 3 (arousal)					
Condition	Arousal	0.56	0.26	2.15	0.031*
Arousal	Δ HR mean (beats/min)	− 0.12	0.14	− 0.86	0.389
Condition	Δ HR mean (beats/min)	1.75	0.64	2.72	0.007**
Model 4 (valence)					
Condition	Valence	0.74	0.33	2.28	0.022*
Valence	Δ HR mean (beats/min)	0.14	0.08	1.65	0.099
Condition	Δ HR mean (beats/min)	1.54	0.64	2.34	0.016*

The participants rated their subjective experience directly after each performance. The Δ HR mean, adjusted for the baseline measurement, was used for this analysis. Appreciation was rated on a 10-point Likert scale. Valence and arousal were rated via the SAM questionnaire with a 9-point Likert scale.

Condition: 0 = Livestream, 1 = Live.

HR, heart rate; SE, standard error.

*p < 0.05; **p < 0.01; ***p < 0.001.

## Discussion

This randomized controlled pilot study aimed to compare the effects of live versus livestreamed concerts—specifically in relation to the physical co-presence of performing musicians—on physiological (HR and HRV) and psychological well-being. Participants in the live condition reported higher levels of appreciation, kama muta (“being moved”), valence and arousal compared to those in the livestream condition. Additionally, the mean HR was significantly higher in the live condition, while accounting for the sequence order of the consecutive measurements (baseline and six music performances). Contrary to our expectations, HRV did not increase in the live group. Altogether, the “liveness” of the music significantly influenced both psychological (appreciation, kama muta, valence and arousal) and physiological (HR mean) responses. However, psychological variables did not mediate the effect on mean HR.

Attending a (live) concert can influence various psychological and physiological responses such as kama muta (the feeling of “being moved”), HR and hormone levels^13,21,41^. Previous research has shown that “liveness” consists of various aspects, meaning that participants’ responses are typically shaped by a combination of physical co-presence with musicians and (other) audience members, as well as the simultaneity of the experience (i.e., the performance unfolding in the moment)^13,15,16^. However, no research has yet focused on how musicians’ physical presence alone shapes the audience’s psychological and physiological responses. In this study, we demonstrate that the condition—i.e. watching the musicians live versus livestreamed, in both cases with audiences physically co-present—influenced several parameters. Regardless of music genre and level of musical accessibility, the live group had a higher mean HR. Participants in the live group reported greater appreciation, kama muta and emotional valence, which could explain the higher HR, as a systematic review has shown that pleasant music tends to cause higher HR compared to unpleasant music^48^. Hence, it is possible that the difference in mean HR between the live and livestream groups was driven by the pleasantness experienced by the audience members. However, the mediation analyses performed in this study did not reveal significant effects of appreciation and valence on mean HR. This could lead us to the assumption that other factors could have been responsible for the higher HR, such as body movement. Participants in the live group may have moved their bodies more to the music, as also shown in previous research, which could have resulted in higher HR^15,40^. To gain deeper insights into the underlying mechanisms and disentangle the effects of HR and body movement, future research should measure both simultaneously and examine whether HR changes primarily reflect emotional responses mediated by the autonomic nervous system or are driven by movement-related physiological demands. Importantly, aside from the “liveness” factor, the type of music was identical, which can also influence HR and HRV and may help explain the heterogeneous results reported in the literature^26,49^. Despite evidence suggesting a mutual relationship between psychological and physiological well-being, this was not the case in this study^45,46^. This may suggest that the increase in HR in the live condition is not merely a function of enhanced subjective experience but may instead reflect additional contextual elements or more complex underlying mechanisms. The observed increase in HR, along with changes in aesthetic appreciation and emotions (kama muta, arousal, valence), can also be explained through embodied music cognition theory, which offers a framework for the complex interplay of cognition, emotion and embodiment, and highlights that participants’ responses are shaped by interaction with the music rather than by one-sided perception^37^. Further research combining this theoretical framework with empirical measures of cognitive and embodied responses could provide deeper insights into how these processes influence live music reception.

Studies on live music show considerable variability in study design and how “liveness” compared to a mediated condition is defined. For example, one can watch a concert on a cinematic screen either alone or with others sharing the same space^5,15,21^. In our study, participants watched a simultaneous cinematic livestream in a concert hall within the same building, where the live performance simultaneously took place. Our results indicate that this difference affects psychological and physiological responses. This effect could be due to participants in the live condition feeling more engaged with the music. Conversely, those in the livestream group might have felt less engaged with the music and musicians, possibly leading to disappointment from not experiencing the live music setting. While participants were randomly assigned to either the live or livestream group, this setup does not fully reflect how people typically engage with concerts, where they for the most part have the autonomy to choose how to engage (e.g., attending a live concert or watching a concert film at the cinemas). Considering participants’ expectations, as well as potential effects introduced through group assignment and experimental setup, could provide valuable insights into this dynamic in future studies. Previous research has shown that the combination of physical co-presence with musicians and other audience members (e.g. concert hall vs. livestream via YouTube) can influence listeners’ perceptions^16^. However, this comparison does not differentiate whether the effect is driven by the physical co-presence of other audience members and/or the musicians. Our findings demonstrate that whether performing musicians are watched live or streamed is an independent factor influencing sociopsychological and physiological responses. In this context, it is important to distinguish physical co-presence from social presence, as the latter may also have influenced participants’ perceptions in both the live and livestream groups. This component was also reflected in the kama muta scale, which captures socioemotional responses through items assessing the extent to which audience members felt connected to other audience members and the musicians, as well as items reflecting broader socio-emotional engagement. As participants in the live group reported higher kama muta scores compared to the livestream group, this suggests that the different levels of physical co-presence in this study also translated into variations in perceived social presence.

In a substantially different study setting, Trost et al. investigated “liveness” through one-on-one performances while participants were examined using functional magnetic resonance imaging (fMRI), providing interesting insights into its underlying neurobiological underpinnings^5^. In this context, sharing the same space with the musician, who could adapt the music based on neurofeedback, showed changes in signals related to emotion processing and the amygdala. Another study on head movement compared a live condition with a recorded setting, with audience members sharing the same space in both conditions^15^. However, in that study, the visual component of watching the musician perform and the simultaneity of the performance differed between the groups. In our study, both conditions occurred at the exact same time (i.e. simultaneity) and both groups were able to watch the musicians perform (either directly or via a cinematic stream), excluding these factors as variables. The absence of a visual component could particularly cause audience members to listen to the music differently. The simultaneity of the concert might also explain the differences in HR response as compared to the study by Shoda et al., which reported higher parasympathetic tone and slower HR in the live condition, in contrast to a recording 10 weeks later^13^. Considering these factors and conceptualizing “liveness” as a continuum of spatial and temporal co-presence is crucial for a nuanced understanding of live music reception. Importantly, the definition of liveness is not static but shaped by complex and dynamic processes embedded in cultural and social contexts^33^. The two states compared in this study—live and livestreamed—are closely tied to notions of authenticity and common practices of (live) music consumption. Especially in today’s increasingly digital landscape, these experiences are influenced by technological advancements and emerging formats, including streaming innovations and virtual/augmented reality, which may further transform perceptions of liveness.

Previous studies have shown positive effects on well-being from various forms of music, such as listening to prerecorded music in healthcare settings, daily music listening and live performances^4,18,58^. However, measuring psychological and physiological well-being remains challenging because of its multidimensional nature and varying definitions^44^. Our observations indicate that the “liveness” of sharing the same space as musicians can significantly benefit psychological well-being, as evidenced by higher valence, appreciation and kama muta scores in the live condition, compared to the livestream. Moreover, participants reported higher emotional ratings on every dimension measured. Notably, the livestream group also showed increased emotional arousal, suggesting that both live and livestreamed concerts fostered a sense of immersion. Immersion is a key component of the flow state—a condition of deep absorption and enjoyment in an activity^59^. Viewing the results through the lens of flow theory could help explain the heightened emotions, and adding a validated instrument such as the Flow State Scale would allow researchers to examine this relationship more directly. The mean HR may suggest that participants were more engaged in the live condition, although it remains overall unclear how the physiological response is affected, as no difference in HRV was observed, which could also be attributed to the pilot setup and methodology of the study. Therefore, attending a concert and being physically present with the musicians can be beneficial, particularly in terms of psychological well-being. Since this study focused on a shorter period, it remains unclear how meaningful this effect is for overall well-being after the concerts. Studying long-term outcomes and incorporating validated well-being measures, such as Seligman’s PERMA model—which assesses positive emotion, engagement, relationships, meaning and accomplishment—could provide valuable insights into the effects on different facets of well-being^60^. This approach may help identify which specific aspects are most affected by live music and cultural participation, supporting more robust conclusions that could guide policymakers and professionals in the cultural sector.

This study has several limitations that should be considered. First, the participants were students from an Arts and Culture study program, making the sample relatively homogenous in terms of age, education level and cultural capital, which limits the generalizability of our findings. Broader recruitment strategies that include participants of diverse sociocultural background and age groups are needed to draw conclusions that are more representative of the general population. Second, this pilot study measured HR and HRV simultaneously in a larger sample of 130 subjects, a challenging technological and logistical feat, which resulted in missing data and may have reduced statistical power. Although there was a trend toward increased cvSDNN (indicating higher parasympathetic activity) in the live condition, this was not observed in the cvRMSSD. The consecutive time intervals may have been too short to register differences in HRV sufficiently, and longer measurement periods (> 10 min) might be promising for future research^22^. Additionally, given our focus on the psychological response’s effect on the physical response in the mediation analysis, it would be valuable to also explore how HR and HRV can, in turn, influence self-reported psychological responses. This could deepen our understanding of live music reception. Third, while we included both pop and classical music of different accessibility levels, we only observed different responses between the live and livestream conditions, irrespective of musical genre or accessibility. However, the fixed sequence of performances limited our ability to isolate the effects of genre and accessibility. Given that both music characteristics are known to influence musical engagement and emotional response, future studies should explore their interaction with performance modality. Last, all selected music pieces were less familiar to the general public to minimize preexisting associations and ensure a comparable level of (un)familiarity across participants. Research indicates that familiar music can elicit stronger psychological and physiological responses^27^, emphasizing the need to consider familiarity alongside other music characteristics (e.g., genre, features, musical accessibility) in future studies on live music reception.

## Conclusion

The “liveness” of the music concerts—here defined as physical co-presence of performing musicians—influenced audience psychological well-being and heart rate (HR). Participants in the live condition reported greater appreciation, kama muta (“being moved”), valence and arousal, and had a higher mean HR compared to the livestream. This suggests that even subtle variations in performance context—such as the physical presence of musicians—can meaningfully affect audience experience. As “liveness” represents also a socially and culturally defined concept with diverse features and subtle variations, further research is needed to identify which specific factors drive certain responses. Understanding this complex interplay of cognitive perception, emotion and embodiment can guide practical applications of “liveness” in everyday and clinical contexts, thereby fostering well-being and public health in a preventive manner. Although this study demonstrates that greater “liveness” can effectively evoke immediate positive responses, more in-depth investigation is needed. By incorporating additional proxies for well-being and examining gradual effects in a demographically representative sample, future research could yield more comprehensive insights.

## Supplementary Information

Below is the link to the electronic supplementary material.

Supplementary Material 1

## Data Availability

The data of the article are not publicly available. The data are available upon reasonable request from the corresponding author.
