# Supplementary material for: Musician presence and its effects on physiological and psychological well-being in live versus livestreamed concerts
Source: Sci Rep. 2026 Feb 9;16:7889. doi: 10.1038/s41598-026-38194-3 (PMC12953899; doi:10.1038/s41598-026-38194-3)
Supplement: Supplementary file 1 — Supplementary Material 1 [file 41598_2026_38194_MOESM1_ESM.pdf]

SUPPLEMENTARY DATA

Supplementary Table 1 CONSORT 2025 checklist

Section/topic	No	CONSORT 2025 checklist item description	Reported on page no.
**Title and abstract**			
Title and structured abstract	1a	Identification as a randomised trial	2
	1b	Structured summary of the trial design, methods, results, and conclusions	2
**Open science**			
Trial registration	2	Name of trial registry, identifying number (with URL) and date of registration	NA
Protocol and statistical analysis plan	3	Where the trial protocol and statistical analysis plan can be accessed	NA
Data sharing	4	Where and how the individual de-identified participant data (including data dictionary), statistical code and any other materials can be accessed	NA
Funding and conflicts of interest	5a	Sources of funding and other support (eg, supply of drugs), and role of funders in the design, conduct, analysis and reporting of the trial	22
	5b	Financial and other conflicts of interest of the manuscript authors	22
**Introduction**			
Background and rationale	6	Scientific background and rationale	3-5
Objectives	7	Specific objectives related to benefits and harms	5
**Methods**			
Patient and public involvement	8	Details of patient or public involvement in the design, conduct and reporting of the trial	NA
Trial design	9	Description of trial design including type of trial (eg, parallel group, crossover), allocation ratio, and framework (eg, superiority, equivalence, non-inferiority, exploratory)	5,6
Changes to trial protocol	10	Important changes to the trial after it commenced including any outcomes or analyses that were not prespecified, with reason	NA
Trial setting	11	Settings (eg, community, hospital) and locations (eg, countries, sites) where the trial was conducted	5
Eligibility criteria	12a	Eligibility criteria for participants	5,6
	12b	If applicable, eligibility criteria for sites and for individuals delivering the interventions (eg, surgeons, physiotherapists)	NA
Intervention and comparator	13	Intervention and comparator with sufficient details to allow replication. If relevant, where additional materials describing the intervention and comparator (eg, intervention manual) can be accessed	6,7

Outcomes	14	Prespecified primary and secondary outcomes, including the specific measurement variable (eg, systolic blood pressure), analysis metric (eg, change from baseline, final value, time to event), method of aggregation (eg, median, proportion), and time point for each outcome	7-9
Harms	15	How harms were defined and assessed (eg, systematically, non-systematically)	NA
Sample size	16a	How sample size was determined, including all assumptions supporting the sample size calculation	5
	16b	Explanation of any interim analyses and stopping guidelines	NA
Randomisation:			
Sequence generation	17a	Who generated the random allocation sequence and the method used	6
	17b	Type of randomisation and details of any restriction (eg, stratification, blocking and block size)	6
Allocation concealment mechanism	18	Mechanism used to implement the random allocation sequence (eg, central computer/telephone; sequentially numbered, opaque, sealed containers), describing any steps to conceal the sequence until interventions were assigned	6
Implementation	19	Whether the personnel who enrolled and those who assigned participants to the interventions had access to the random allocation sequence	6
Blinding	20a	Who was blinded after assignment to interventions (eg, participants, care providers, outcome assessors, data analysts)	6
	20b	If blinded, how blinding was achieved and description of the similarity of interventions	NA
Statistical methods	21a	Statistical methods used to compare groups for primary and secondary outcomes, including harms	9
	21b	Definition of who is included in each analysis (eg, all randomised participants), and in which group	10
	21c	How missing data were handled in the analysis	9
	21d	Methods for any additional analyses (eg, subgroup and sensitivity analyses), distinguishing prespecified from post hoc	NA
**Results**			
Participant flow, including flow diagram	22a	For each group, the numbers of participants who were randomly assigned, received intended intervention, and were analysed for the primary outcome	10
	22b	For each group, losses and exclusions after randomisation, together with reasons	10
Recruitment	23a	Dates defining the periods of recruitment and follow-up for outcomes of benefits and harms	5
	23b	If relevant, why the trial ended or was stopped	NA
Intervention and comparator delivery	24a	Intervention and comparator as they were actually administered (eg, where appropriate, who delivered the intervention/comparator, how participants adhered, whether they were delivered as intended (fidelity))	7
	24b	Concomitant care received during the trial for each group	NA
Baseline data	25	A table showing baseline demographic and clinical characteristics for each group	10, Table 2
Numbers analysed, outcomes and estimation	26	For each primary and secondary outcome, by group:<br>● the number of participants included in the analysis<br>● the number of participants with available data at the outcome time point<br>● result for each group, and the estimated effect size and its precision (such as 95% confidence interval)<br>● for binary outcomes, presentation of both absolute and relative effect size	10-12
Harms	27	All harms or unintended events in each group	NA

Ancillary analyses	28	Any other analyses performed, including subgroup and sensitivity analyses, distinguishing pre-specified from post hoc	NA
**Discussion**			
Interpretation	29	Interpretation consistent with results, balancing benefits and harms, and considering other relevant evidence	13-15
Limitations	30	Trial limitations, addressing sources of potential bias, imprecision, generalisability, and, if relevant, multiplicity of analyses	16

Citation: Hopewell S, Chan AW, Collins GS, Hróbjartsson A, Moher D, Schulz KF, et al. CONSORT 2025 Statement: updated guideline for reporting randomised trials. BMJ. 2025; 388:e081123.
https://dx.doi.org/10.1136/bmj-2024-081123
© 2025 Hopewell et al.

Supplementary Table 2 Kama muta items

**Six of these statements addressed the extent to which the following sensations, feelings or actions were experienced during or right after the music:**
Tears
Chills or shivers
A warm feeling in the center of the chest
Choked up
Refreshed, energized or exhilarated
Desire to move your body such as toe-tapping, head-bobbing or dancing.
**The remaining items were statements on social emotions for which participants could indicate their agreement:**
I felt like telling someone how much I care about them
I wanted to hug someone
I felt moved/touched
I felt connected to the performers
I felt connected to the other audience members
I felt like I shared emotions with other audience members.

The kama muta scale, which conceptualizes the feeling of 'being moved', was used in this study with twelve items on a 5-point Likert scale (Cronbach's $\alpha = 0.91$), which is based on the validated KAMMUS-S and KAMMUS-2 measures.

Supplementary Table 3 Linear mixed model of psychological parameters

	Parameter	Intercept	Sequence of music performances	Condition (0=Livestream, 1=Live)
Appreciation (10-point Likert scale)	β (95% CI)	7.7 (7.3; 8.1)	-0.4 (-0.5; -0.3)	0.7 (0.27; 1.07)
	p-value	<0.001***	<0.001***	0.001**
Kama muta (5-point Likert scale)	β (95% CI)	1.9 (1.8; 2.1)	-0.1 (-0.1; -0.0)	0.2 (0.3; 0.6)
	p-value	<0.001***	<0.001***	<0.001***
Arousal (9-point Likert scale)	β (95% CI)	6.3 (6.0; 6.5)	-0.1 (-0.2; -0.1)	0.6 (0.3; 0.9)
	p-value	<0.001***	<0.001***	<0.001***
Valence (9-point Likert scale)	β (95% CI)	4.0 (3.59; 4.34)	0.1 (0.0; 0.1)	0.6 (0.1; 1.0)
	p-value	<0.001***	0.015*	0.011*

Linear mixed models were conducted for all the listed psychological parameters while considering the condition (0=Livestream, 1=Live) and the sequence order of the baseline measurement and six music performances. Based on information criteria (AIC and BIC) and log-likelihood test, a random slope for the sequence order was included in the model. The kama muta scale is based on 12 items (5-point Likert), which were averaged.

Abbreviations: AIC= Akaike Information Criterion; BIC= Bayesian Information Criterion.

*p<0.05; **p<0.01; ***p<0.001.

Supplementary Figure 1 Ground plan of the concert venue

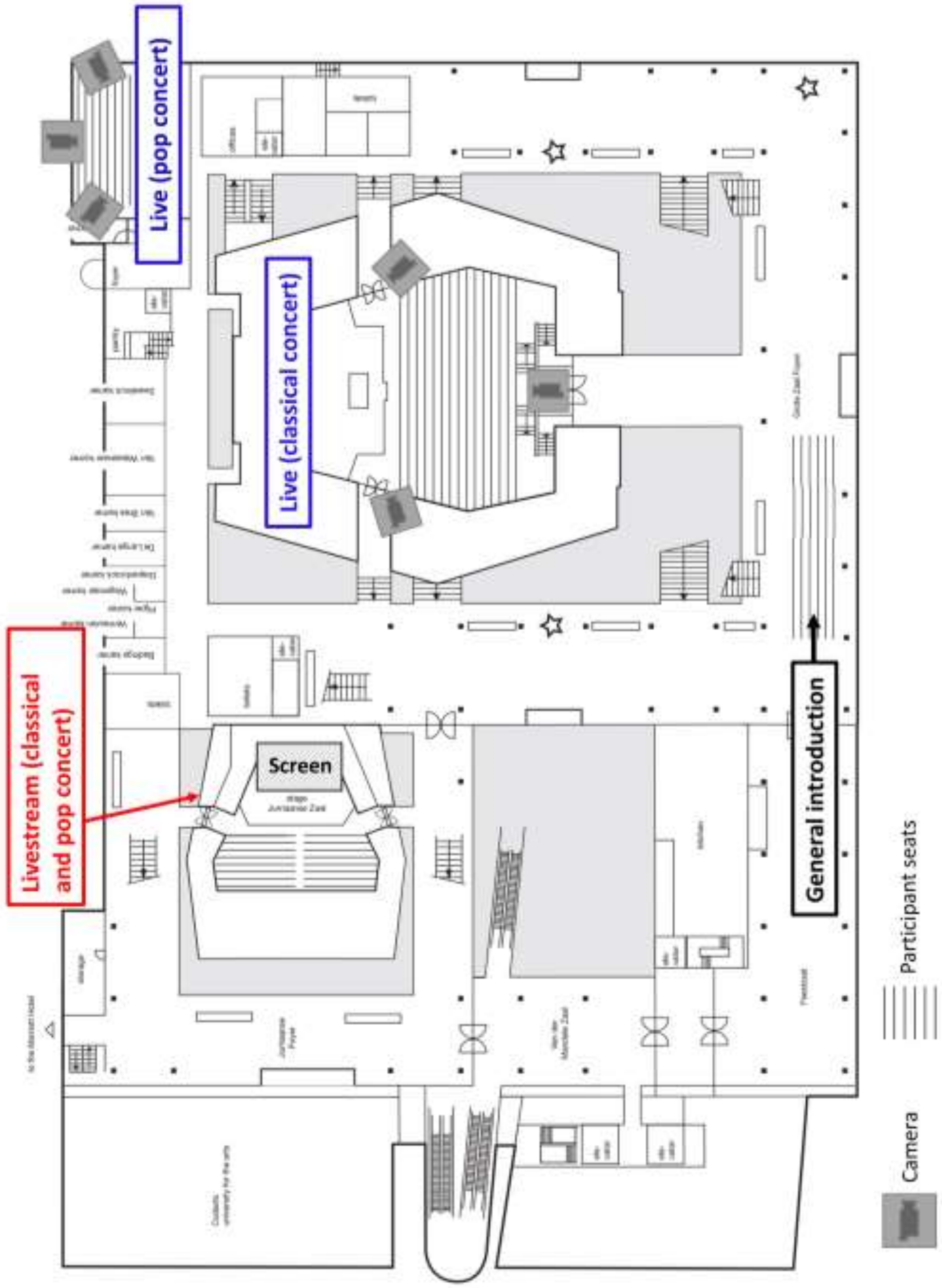

The figure depicts the ground plan of the concert venue 'De Doelen' in Rotterdam. First, participants were seated for the general introduction, where the baseline measurement took place. Subsequently, the randomization allocation was revealed, and participants entered their respective concert hall (livestream group in red and live group in blue). Between the two concerts (classical and pop), there was a 15-minute break during which all participants were instructed to stand up and move for comparability reasons. The live group changed rooms due to prior equipment installation by the musicians, while the livestream group was seated again in the same room.
